# A multi-layer soft lattice based model for Chinese clinical named entity recognition

**DOI:** 10.1186/s12911-022-01924-4

**Published:** 2022-07-30

**Authors:** Shuli Guo, Wentao Yang, Lina Han, Xiaowei Song, Guowei Wang

**Affiliations:** 1 State Key Laboratory of Intelligent Control and Decision of Complex Systems, School of Automation, Beijing Institute of Technology, Beijing, China; 2 Department of Cardiology, The Second Medical Center, National Clinical Research Center for Geriatric Diseases, Chinese PLA General Hospital, Beijing, China

**Keywords:** Clinical named entity recognition, Clinical text mining, Fine-tuning BERT, Medical information processing, Transformer, Word-character lattice

## Abstract

**Objective:**

Named entity recognition (NER) is a key and fundamental part of many medical and clinical tasks, including the establishment of a medical knowledge graph, decision-making support, and question answering systems. When extracting entities from electronic health records (EHRs), NER models mostly apply long short-term memory (LSTM) and have surprising performance in clinical NER. However, increasing the depth of the network is often required by these LSTM-based models to capture long-distance dependencies. Therefore, these LSTM-based models that have achieved high accuracy generally require long training times and extensive training data, which has obstructed the adoption of LSTM-based models in clinical scenarios with limited training time.

**Method:**

Inspired by Transformer, we combine Transformer with Soft Term Position Lattice to form soft lattice structure Transformer, which models long-distance dependencies similarly to LSTM. Our model consists of four components: the WordPiece module, the BERT module, the soft lattice structure Transformer module, and the CRF module.

**Result:**

Our experiments demonstrated that this approach increased the F1 by 1–5% in the CCKS NER task compared to other models based on LSTM with CRF and consumed less training time. Additional evaluations showed that lattice structure transformer shows good performance for recognizing long medical terms, abbreviations, and numbers. The proposed model achieve 91.6% f-measure in recognizing long medical terms and 90.36% f-measure in abbreviations, and numbers.

**Conclusions:**

By using soft lattice structure Transformer, the method proposed in this paper captured Chinese words to lattice information, making our model suitable for Chinese clinical medical records. Transformers with Mutilayer soft lattice Chinese word construction can capture potential interactions between Chinese characters and words.

## Introduction

The aim of named entity recognition is to extract entities with actual meaning from massive unstructured text [1]. In the clinical and medical domain, clinical named entity recognition (CNER) recognizes and classifies medical terms in unstructured medical text records, including symptoms, examinations, diseases, drugs, treatments, operations, and body parts. As a combination of structured and unstructured texts, the rapidly growing biomedical literature contains a significant amount of useful biomedical information. Moreover, NER is a key and fundamental part of many natural language processing (NLP) tasks, including the establishment of a knowledge graph, question answering system, and machine translation. Therefore, CNER can extract meaningful medical knowledge to support medical research and treatment decision-making.

Recently, there has been renewed interest in various medical and biomedical tasks, such as protein–protein interaction (PPI) extraction [2], drug-drug interaction (DDI) extraction [3], and chemical-protein interaction (CPI) extraction [4, 5]. The objection of these tasks is to recognize entities and extract possible meaningful relations between them. For example, extracting pivotal knowledge about previously prescribed treatments or drugs and obtaining the relationship between them and diseases can be helpful to support decision-making [6]. Most of these tasks require two steps. First, medical entities mentioned in a given text are recognized by named entity recognition (NER) technologies. Next, the relation of each entity pair is categorized into the group required by tasks [7]. For medical information mining from unstructured text data in clinical records, NER is a crucial step, and the NER results may affect the performance of these tasks.

For the application of medical name recognition, initially, the approaches of establishing well-designed keyword search, rule-based systems [8], and feature sets [9] can usually achieve good performance. Nevertheless, the construction of the list of keywords and rules requires much manual work. Supervised machine learning [10] models have become a way to solve this problem and perform better than unsupervised approaches. However, their performance depends highly on the quality of annotated data.

As deep learning advances, deep learning for NER based on word embedding has gradually become popular. Long short-term memory (LSTM) with a conditional random field (CRF) [11, 12] is a typical deep learning model for NER that can learn similar representations for semantically or functionally similar words and can effectively extract features of text data. Although these deep learning models can significantly reduce resource consumption and manual labeling costs, they have a drawback: the embedding of the same word in varying semantic contexts is identical.

Recently, natural language processing (NLP) has entered the era of pretrained models. Transformer [13], embedding from language models (ELom) [14], and bidirectional encoder representations from transformers (BERT) [15] in many NLP tasks have achieved current state-of-the-art results. The above most recent representation-learning models can learn structural information of language and generate more effective word representations from large-scale unannotated data in model training than those trained only from limited annotated data. Then, these pretrained models are applied to CNER tasks by fine-tuned strategies.

For example, BioBERT [16] is a typical pretrained BERT. In pretraining, PubMed and PubMed Central (PMC) publications were used to train the vanilla BERT. Although BioBERThas a similar architecture to BERT, it contains rich linguistic information about the pretrained biomedical corpus. Thus, BioBERT achieved state-of-the-art results on three biomedical text tasks, including biomedical NER [17]. However, this work cannot encompass the entire language biomedical and clinical entity normalization tasks, especially in languages such as Chinese, without spaces between characters.

While the BERT model can generate a BERT embedding of English and overcome the limitations of traditional embedding that cannot capture the ambiguity, the pretraining of BERT corrupts the input and magnifies the sparsity of the data, and the BERT embedding obtained from training corpora with scarcity is likely to accomplish the tasks well. We review some representative related approaches and previous studies. In the early stages of medical named entity recognition development, the common NER approach was mostly manually crafted expert rules, dictionaries, and heuristics to identify entities of interest. These rule-based approaches are still applied today, but they have a drawback: many rules are required to maintain optimal performance, cannot automatically learn text based on text federation, is vulnerable to data limitations and therefore lacks portability and robustness. Hence, the hidden Markov model (HMM) [18], maximum entropy Markov model (MEMM) [19], and conditional random field (CRF) have become classic CNER methods that can solve sequence labeling problems. However, NER based on traditional machine learning highly relies on feature selection, and suffers from a lack of generalizability. Moreover, their computation was expensive and had data sparsity issues [20].

In recent years, with the increase in computing power, NER based on deep neural networks has been able to solve these problems and is receiving more attention. In particular, the bidirectional LSTM with a CRF layer (BiLSTM-CRF) has less artificial conduction than rule-based methods and is powerful in capturing the potential relationship between the token and the cue. Wang et al. [21] proposed a BiLSTM-CRF BioNER combined multitask learning framework to solve NER. Chen et al. [22] proposed CollaboNet to reduce false positives via multiple LSTM with CRF. Wunnava et al. [23] employed a BiLSTM-CRF to recognize and exact adverse drug events (ADEs) and had excellent extraction accuracy. Zhang et al. [24] proposed Lattice-LSTM by improving the internal structure of LSTM, and Lattice-LSTM directly connected the embedding of the Chinese character and Chinese word according to the correspondence to improve CNER.

These methods based on BiLSTM-CRF focus on feature extraction between words, but they do not have general-purpose priors and need to be trained from the beginning for specific tasks. With the Google BERT pretraining model proposed, this problem was overcome. Several CNER studies [25] used the outputs of the BERT model as word embedding and then input these BERT embeddings into the traditional BiLSTM-CRF model. The advantage of this approach is that it handles lexical ambiguity in combination with context.

Current researchers mainly focused on BERT pretrained on a large clinical unlabeled corpus, which achieved the optimal performance on extracting the named entities and relations of these entity pairs from the encounter notes, operation records, pathology notes, radiology notes, and discharge summaries of patience [26]. Li et al. [27] took a BERT-based model that is easily fine-tuned to normalize the types of entities and clinical terms and outperformed them on the MADE 1.0 corpus compared with other models.

The studies mentioned above all use RNN and LSTM as the core to obtain contextual information to complete the NER task. However, LSTM, as a sequence model, cannot be computed in parallel, resulting in a long training time. Therefore, it is the focus of this study to propose a method that can be computed in parallel and still has the function of LSTM to capture contextual information effectively. Therefore researchers started to focus on the use of CNN in CNER, proposing RD-CNN-CRF [28], ID-CNN [29]. However, CNN need to incorporate more convolutional layers in order to obtain contextual information, leading to many hyperparameters of the network and still requiring longer computation time.

However, most NER methods often take a long time for training and are unable to perform parallel computing. Motivated by the recent success of transformer and BERT approaches in dealing with various NLP tasks, we utilized the following methods in this paper:

1. We introduced a novel model based on pretrained BERT from the Chinese clinical corpus for NER, which enables us to model information about characters and words. Our experimental results show that this method has high accuracy on the NER task.
2. We introduce a selection scheme of word separation result, Soft Term Position Lattice, which can combine lexical information and lexical importance to select lexical location information that is important for the model to locate the entity span.
3. We presented soft lattice structure transformer layers to build a simple and efficient module that can select more correct words and capture the contextual information during NER training.

## Materials and methods

### Datasets

To prove the superiority of our methodology compared to traditional models, we trained and evaluated our method and related baseline model on the 2019 CCKS dataset. As shown in Table 1 there were 6 categories of clinical entities to be identified, including disease, image review, test, treatment, drug, and anatomy in the CCKS-CNER 2019 dataset. We take the first 90% of CCKS2019 data for the training dataset, and the remains are used as the test dataset.

**Table 1:** Proportion of entities in training date

Label	Count
Disease	2116
Image review	222
Test	318
Treatment	765
Drug	456
Anatomy	1486
Total	5363

The entities in Chinese text are represented by “BIO”, where “B”, “I”, and “O” mean the beginning of an entity, Chinese characters that are inside of a Chinese entity, and other unrelated characters, respectively.

### Method

**Fig. 1:**
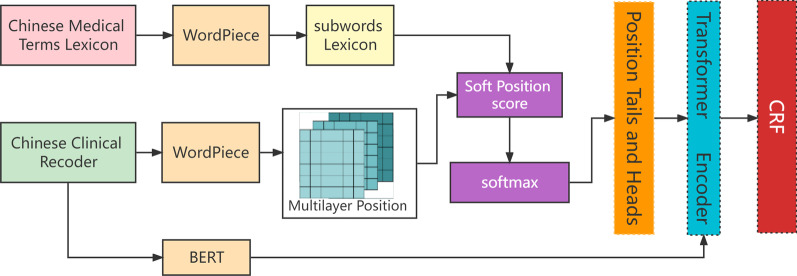
The architecture of our proposed method

We proposed a lattice structure transformer named the entity recognition model that can capture the location of entities accurately by combining sequences of characters with a pretrained model. The architecture of our model is shown in Fig. 1, which consists of four parts: the WordPiece module, the BERT module, the lattice structure Transformer module and the CRF module. An overview of these methods is shown in Fig. 1.

First, the WordPiece module is utilized to process the input text data to obtain the common medical terms and the common sequences of Chinese characters. Based on this work, we establish a medical clinic dictionary and provide the lattice structure transformer layer with position information of texts. Next, the BERT module was applied to convert text into embedding and captured the deep knowledge of the text, including part-of-speech, implicit language rules and major sentence features.

Then, we applied the lattice structure Transformer module to achieve the function of LSTM use based on the character-word lattice structure from the WordPiece module. LSTM uses gating mechanisms to alleviate the gradient problem. The gating mechanism makes the LSTM have more training parameters, resulting in the LSTM requiring an extensive training time. To avoid this problem, we design the soft lattice structure Transformer for learning contextual dependencies and location information.

Finally, the CRF layer was used to legally decode the output of the previous module and learn the constraints of labels. The final output of CRF is the sequence tagging. The specific functions and underlying principles of each module are described in the following sections.

#### WordPiece

There is some difficulty in NLP tasks for Chinese clinical text. Chinese terminologies, English terminologies, English abbreviations and numeric symbols [26] may appear in Chinese clinical text. Clinical Chinese texts mixed with multiple expressions have many long-tail concepts and terminologies, which have complex structure and the variety of phrase combinations. These terminologies have a long sequence of Chinese characters and different sequences that have the same meaning.

Although the smallest unit of Chinese is the word and Chinese sentences cannot be split into letters such as English, the extensive length of terms in the field of clinical medicine makes WordPiece applicable to the field of clinical medicine. These different Chinese sequences pointing to the same thing often have the same sub-sequences, similar to the characteristics of English. These different granularities of subwords all contain features that are close to the original content of the text and can enhance entity boundary information. This is similar to the English language, so we use WordPiece to obtain the subwords and then build a dictionary containing the subwords. This dictionary records the number of subwords and the category of the term.

WordPiece has stronger guarantees that each subword unit has been seen in the training text. It enables the model to try learning the representation of a word out of vocabulary. The primary implementation of WordPiece is the BPE (byte-pair encoding) algorithm. WordPiece essentially is a data-driven approach. It selects adjacent subwords that maximize the probability of the language model being added to the word list and merges the selected adjacent subwords into new subwords, given an evolving vocabulary.

The WordPiece algorithm was first required to confirm the training corpus, and we used D to represent the number of desired tokens. The goal of optimization is to select D WordPieces to minimize the number of WordPieces in the resulting vocabulary when obtaining subwords according to the chosen WordPiece model. WordPiece can achieve the flexibility of Chinese characters in medical recorders. Therefore, we utilized WordPiece to process the various categories of Chinese medical terms in specialist medical corpus Yidu-N7K and obtained a lexicon containing subwords that frequently occurred and subwords' categories. These subwords in this lexicon are applied to perform segmentation on the sentences in train dataset. And this lexicon is the foundation of the lattice structure transformer in “Soft lattice transformer structure” section.

#### BERT embedding

The BERT model is a typical representation learning model, which is pretrained on a large unlabeled text corpus to learn better text features. BERT is often applied to a wide range of tasks by fine-tuning.

There are two main new component models proposed in BERT: the masked language model (MLM) and next sentence prediction (NSP).The MLM masks some of the words at each iteration and is masked at random, and then the model predicts the existence of these masked words given their context. The role of NSP is to obtain relationships between sentences. Sentences A and B are fed into BERT, and [CLS] obtained by BERT is used to predict whether B is the following sentence of A.

In our model, instead of using the traditional word vector, the BERT model uses the attention-based transformer structure to generate embedding as the input. We employed MC-BERT model pretrained on unlabeled Chinese clinical records available from Zhang et al. [30]. As a pre-trained language model comprising a stack of multi-head self-attention layers and fully connected layers, MC-BERT is pre-trained on a large-scale unlabeled corpus of Chinese clinical text, including Chinese community biomedical question answering, Chinese medical encyclopedia, Chinese electronic health records (EHR). And whole entity masking and whole span masking strategies are utilized to pre-train MC-BERT.

**Fig. 2:**
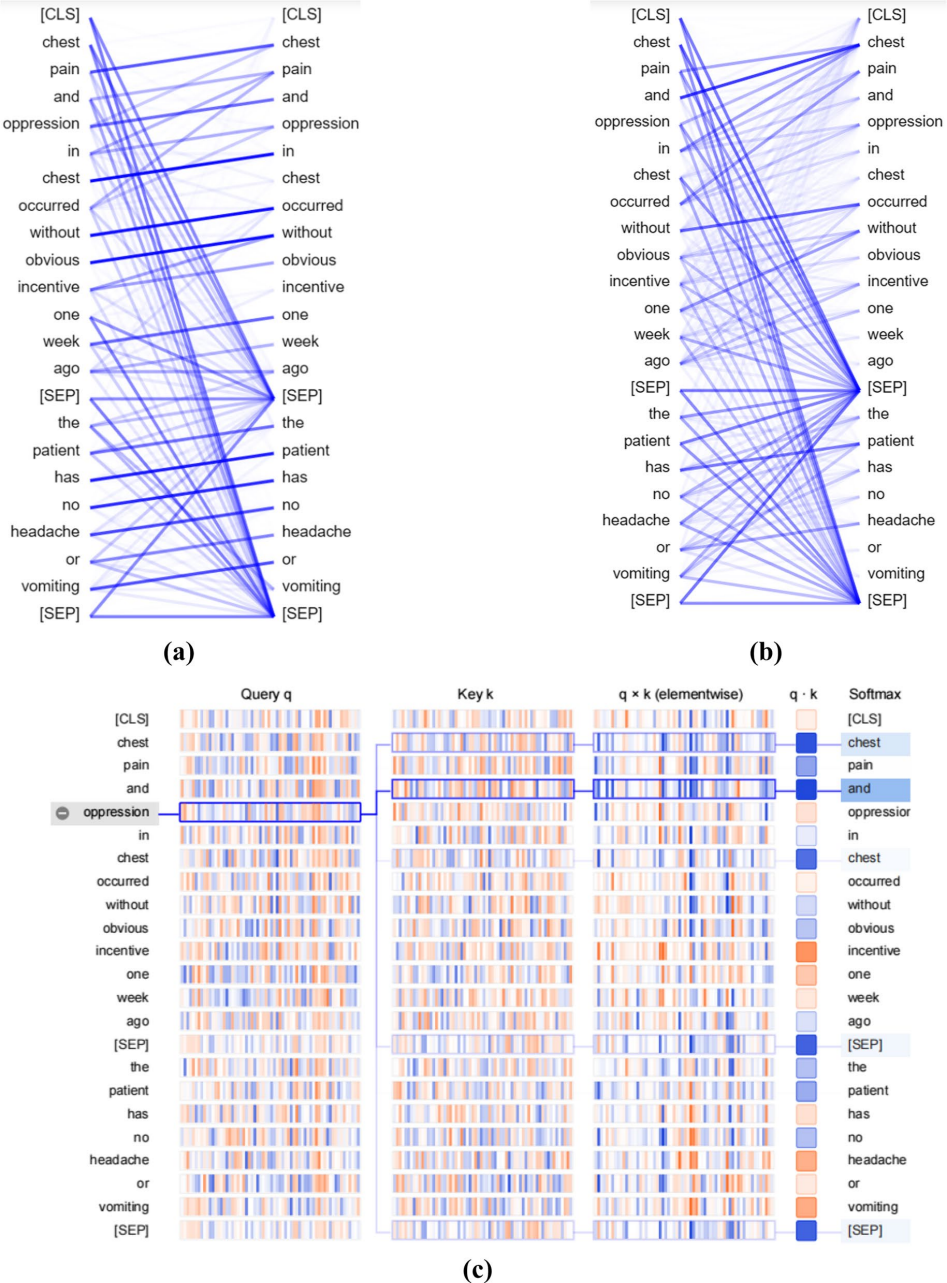
BERT visualization: **a** attention-head view for BERT, for inputs, The left and center figures represent different layers/attention heads. The right figure depicts the same layer/head as the center figure, but with Sentence A → Sentence B filter selected [31]; **b** Model view of BERT, for same inputs, layers 4; **c** Neuron view of BERT for layer 0, head 0

This section shows the visualization of the attention spread of BERT in CNER. The colored squares of Fig. 2c represent one or more attention heads. The words in the picture are the Chinese entities in the clinical recorder to be recognized. The lines in the middle of Fig. 2a and b connect the Chinese character on the left and right, representing the attention score of the tokens. The saturation of color in Fig. 2c represents the strength of attention.

#### Soft lattice transformer structure

The lattice structure has been proved to have a great benefit to recognize Chinese Named Entity. The lattice structure is capable of capturing dependencies between characters and matched lexical words and enhancing the ability of capturing useful local context. In this section, we obtained the lattice by splitting a sentence according to the lexicon from WordPiece.

In lattice structure Transformer, the spans in the lattice structure Transformer lattice have the head and tail of subwords in the lexicon, which can reinforce entity boundaries. For the position encoding of spans, abandoning the absolute position encoding, we construct head position encoding and tail position encoding by relative position encoding [32].

**Fig. 3:**
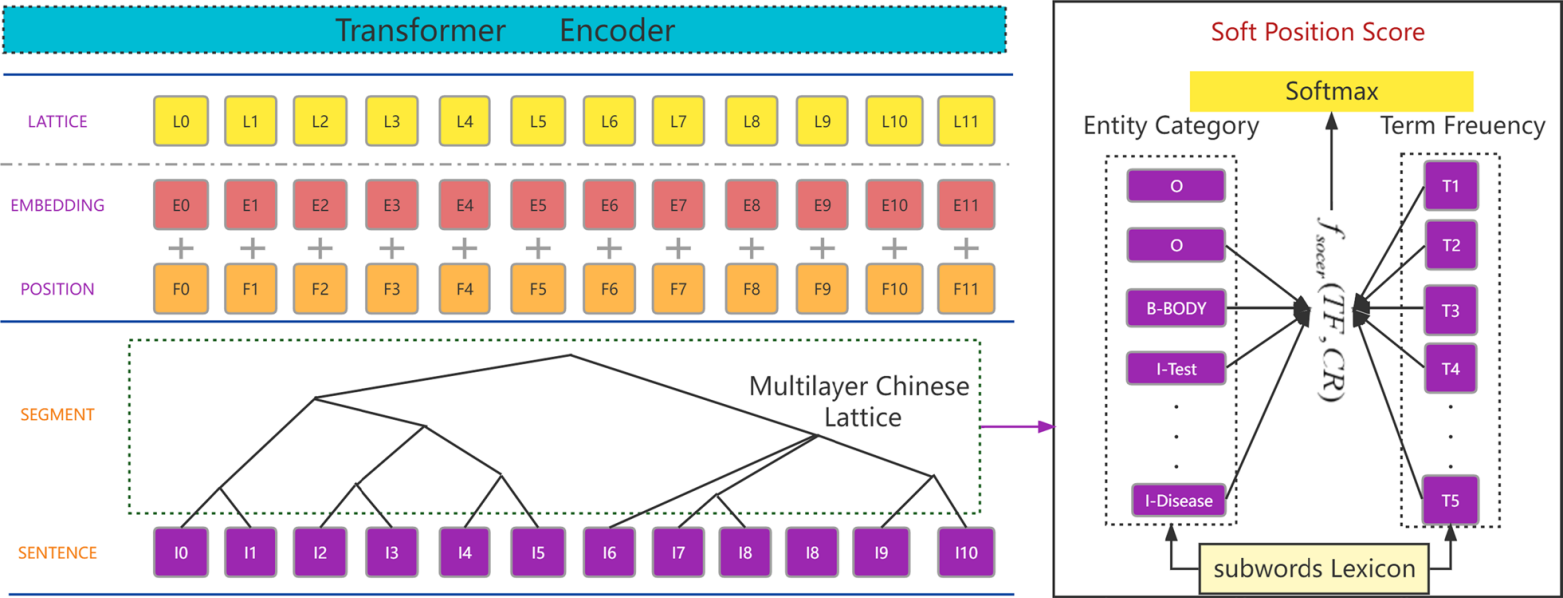
Soft Lattice Chinese Transformer structure

Combined with the well-designed position encoding, relative position encoding of span introduced lexical information into the transformer without loss. Hence, the lattice structure transformer can fully leverage the lattice information and improve the performance of CNER. The Transformer encoder in the lattice structure Transformer layer requires spans with different lengths of Chinese words to encode the interactions in these words. Instead of directly employing positional encoding adapted to the standard Transformer, we use relative position embedding [29] to model the position encoding of spans and the positional difference (Fig. 3).

We design the word-character lattice model the same way as the Lattice BERT [33], where we first split the sentences according to the lexicon in WordPiece to obtain a sequence of subwords of the sentences. Then we get a sequence of characters in Chinese sentences in character units. Since the Transformer requires positional information, the word-character lattice is represented in position by assigning positional indexes. Finally, we transform the position lattice and the position representation into a flat structure [32].

There may be multiple layers of Chinese sentence segmentation results. Each layer of segmentation results is subwords of different granularity. The importance of different subword results for the model to locate the entity span varies. The choice of which subword results among the multi-layer subword results determines the performance of the final entity recognition.

**Fig. 4:**
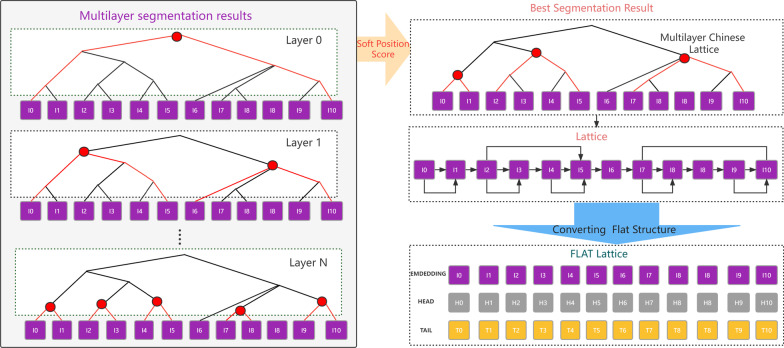
Multiple layers of Chinese sentence segmentation

We form multiple layers of Chinese sentence segmentation results according to their granularity. Each layer has a different level of granularity. As shown in Fig. 4, the sentence in the first layer is a whole and has the greatest granularity. In the second layer, the segmentation is divided into two parts, which have different levels of granularity. We then calculate the TF-CR score of every layer to get the result of the largest score. For example, in Fig. 1, the best splitting result contains subwords with different granularity. Finally, the result is transformed into the flat lattice.

The aim of Soft Position Score is to selecte splitting results. The choice of which word separation result determines the performance of the final entity recognition. For this problem, the scores of each subword result are obtained by using the categories and word frequencies to which the words in the subword dictionary belong, and the subword result with the largest score is selected by softmax.

1 $$f_{socer}^{i} (TF,CR) = TF \times CR = \sum\limits_{i = 0}^{L} {\left( {\frac{{\left| {x^{i} } \right|}}{{N_{c}^{i} }}*\frac{{\left| {x^{i} } \right|}}{x}} \right)}$$

TF, CR reprents respectively term frequency and category ratio. $TF = \frac{{\left| {x^{i} } \right|}}{{N_{c}^{i} }}$ where $x^{i}$ is the number of occurrences of subword $i$, and $N_{c}^{i}$ is total number of subword $i$ in category $c$. $CR = \frac{{\left| {x^{i} } \right|}}{x}$ where $x$ denotes the number of occurrences of across all categories.

Once the subword results have been determined, the subwords are transformed into positional information and the lexical information is fused using the positional information. This ensures that pre-trained word embedding models can be used, while minimising the loss of information due to lexical matching errors. Most importantly, this approach eliminates the need to introduce lexical information in terms of word embedding, allowing the advantages of parallel computing of the model to be fully exploited and increasing the speed of model inference.

As shown in Fig. 3, the input to the model is a character sequence, together with all character subsequences selected by Soft Term Position Lattice. $w_{d} (b,e)$ is used to denote such a subsequence that begins with character index b and ends with character index e.

2 $$x_{b,e}^{d} = embed(w_{d} (b,e))$$

3 $$d_{b,e} = e - b$$

where $embed$ denotes the same word embedding lookup table. $d_{b,e}$ denotes the length between the head and tail of the word. Then, we use sine and cosine functions of different frequencies to encode position spans.

4 $$P_{d}^{2k} = \sin (d_{b,e} /10000^{{2k/d_{\bmod el} }} )$$

5 $$P_{d}^{2k + 1} = \cos (d_{b,e} /10000^{{2k/d_{\bmod el} }} )$$

6 $$R_{b,e} = {\text{Re}} LU(W_{r} P_{d} )$$

where $R_{b,e}$ is the final relative position [30] encoding of spans and $W_{r}$ is a learnable parameter.

7 $$A_{b,e} = E_{{x_{i} }}^{T} W_{q}^{T} W_{k,E} E_{{x_{j} }} + E_{{x_{i} }}^{T} W_{q}^{T} W_{k,R} R_{b,e} + u^{T} W_{k,E} E_{{x_{j} }} + v^{T} W_{k,R} R_{b,e}$$

We replace $A$ with $A_{b,e}$ in Eq. (8). The following calculation is the same as that of the vanilla Transformer, where $W_{q}^{{}}$, $W_{k,E}$, $W_{k,R}$, $u$, and $v$ are learnable parameters. Equipping the above proposed relative positional embedding, the hidden state can be computed as follows:

8 $$h_{i}^{l} = Trm(h_{i}^{l - 1} ,A_{i,b,e}^{l - 1} )$$

where $h_{i}^{l}$ and $Trm$ denote the $i$th hidden state and represent the transformation function.

#### CRF

The NER is essentially a sequence labeling task. Since labels are not independent of each other, it is essential to thoroughly learn the internal relationship of tags according to the labeled dataset. As a conditional probability model, CRF is able to handle contextual feature information and effectively consider constraints among tags to reduce illogical sequences. Finally, we used the Viterbi algorithm to find the optimal path achieving the maximum probability in the prediction process.

Given the transition score matrix $A$ and $p_{i,j}$ in matrix $P$ from the lattice structure Transformer layer, representing the probability of the label $i$ of the word $j$, CRF can calculate the score of the sequence. denotes the sentence sequence. $y = \{ y_{1} ,..,y_{i} ,..,y_{n} \}$ is an output sequence of the tag; thus, the corresponding scoring function is computed as follows:

9 $$score(x,y) = \sum\limits_{i - 1}^{n} {p_{{i,y_{i} }} } + \sum\limits_{i - 1}^{n + 1} {A_{{y_{i} ,y_{i + 1} }} }$$

where $A_{{y_{i} ,y_{i + 1} }}$ represents the transition probability from $y_{i}$ to $y_{i + 1}$. Then, we can obtain the normalized probability by the softmax function.

10 $$p(y|x) = \frac{\exp (score(x,y))}{{\sum\nolimits_{y} {\exp (score(x,y)} }}$$

The objective function and optimized object of CRF can be computed schematically by

11 $$y^{*} = \arg \max (score(x,y^{\prime}))$$

### Experimental setup

There are several sentences in each medical record of the annotated Chinese medical record data, which would result in an excessive sample if we did not divide the whole record. To restrict the sentence length, therefore, according to Chinese punctuation, we separated each record and treated each Chinese sentence in each record as a single sample. The first layer of the model, the embedding layer, turns characters or words into vector representations. Based on 300-dimensional pretrained glove Chinese character embedding pretrained on wiki text, each Chinese character can be mapped to an embedding by unsupervised learning.

Our model has the following number of parameters based on the transformer: one self-attention layer with 512 input and output dimensions and 8 attention heads. We trained the language models with SGD. The initial learning rate of 6e−4 and coefficients of epoch is 100, batch is 10, early stop is 25 to avoid overfitting; the learning rate is set to warm up over the first 50 k steps, and the learning rate exhibits linear decay. We used a dropout probability of 0.1 on all layers.

### Evaluation methodology

We evaluated the overall precision (P), recall (R) and F1-score (F1) based on official strict matching. P, R and F1 can help us eliminate the effects of unbalanced data itself and to fully predict the result.

## Results

The results of the soft lattice structure Transformer and baselines on the CCKS 2019 dataset are shown in Table 2. The baseline model, LSTM-CRF, is shown in the first row. The second row displays GRU with a CRF layer. The third row is the BERT with a CRF as a baseline model. In the fourth and fifth rows in Table 1, the performance obtained by BERT-LSTM-CRF and BERT-GRU-CRF, respectively, is superior to that of the baselines without BERT, which further demonstrated the effectiveness of BERT.

**Table 2:** Test results on the CCKS2019 dataset

Models	P	R	F1	Cost time
LSTM-CRF	79.32	83.21	80.13	11 h
GRU-CRF	80.23	82.03	82.14	9 h 7 m
BERT-CRF	83.47	80.14	82.50	7 h 45 m
BERT-LSTM-CRF	86.07	86.23	86.71	8 h 32 m
BERT-GRU-CRF	85.35	86.18	85.36	7 h 23 m
BERT-FLAT-CRF[32]	86.56	86.56	87.49	6 h 6 m
BERT-Soft Lattice structure Transformer-CRF	87.86	87.53	87.83	6 h 15 m

In the last two rows, the soft lattice structure Transformer achieved an F1 of 0.87, and the lattice structure Transformer realized an F1 of 0.86 F1. Augmented with the soft lattice structure Transformer model, the model achieved a precision of 0.876, a recall of 0.875, an F-measure of 0.878 and a training time of 6.25 h. The results show that our model achieves the performance of the BERT-LSTM-CRF model. While the results show small (almost negligible) improvements, the performance in terms of time cost is less than that of the baseline model. Overall, the results illustrate our method’s superior predictive performance in CNER.

**Fig. 5:**
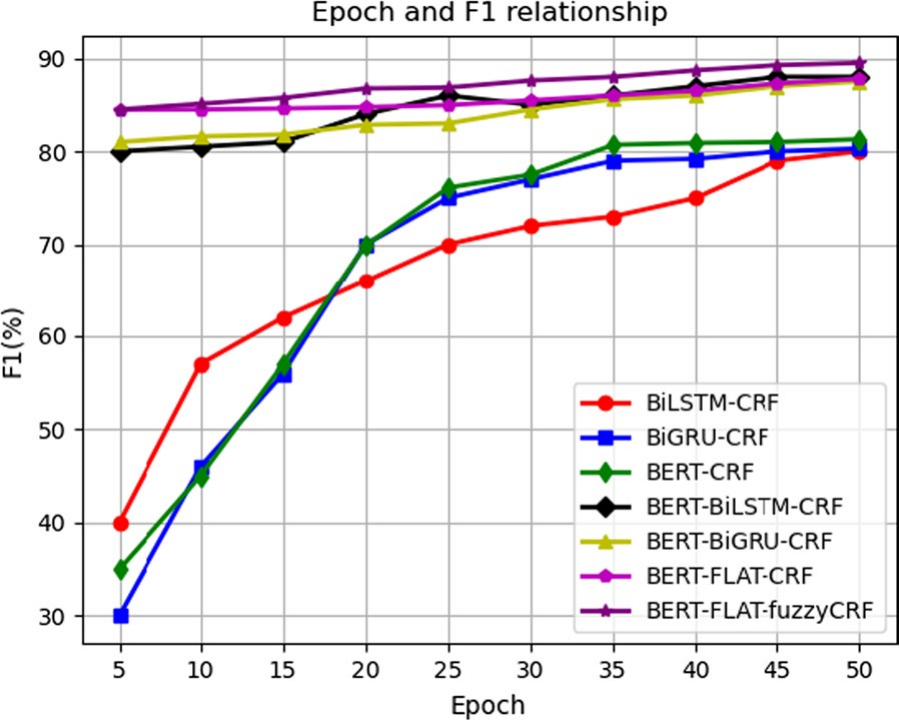
Comparison of the Epoch and F1 relationship of different models

The overall trends of epoch and F1 are provided in Fig. 5. We analyzed the relationship between epoch and F1 in the four models. The F1 score of the NER model without BERT gradually improves from a lower point to the optimal score, while the F1 score of the model with BERT remains at a higher level, and it takes fewer times to reach the optimal F1 score. In addition, compared to the NER model based on BiLSTM or BiGRU, the model is better. Table 2 provides the time required to train each model. From Table 2, it is easy to conclude that the BERT-GRU-CRF model has the shortest training time of the NER models.

**Fig. 6:**
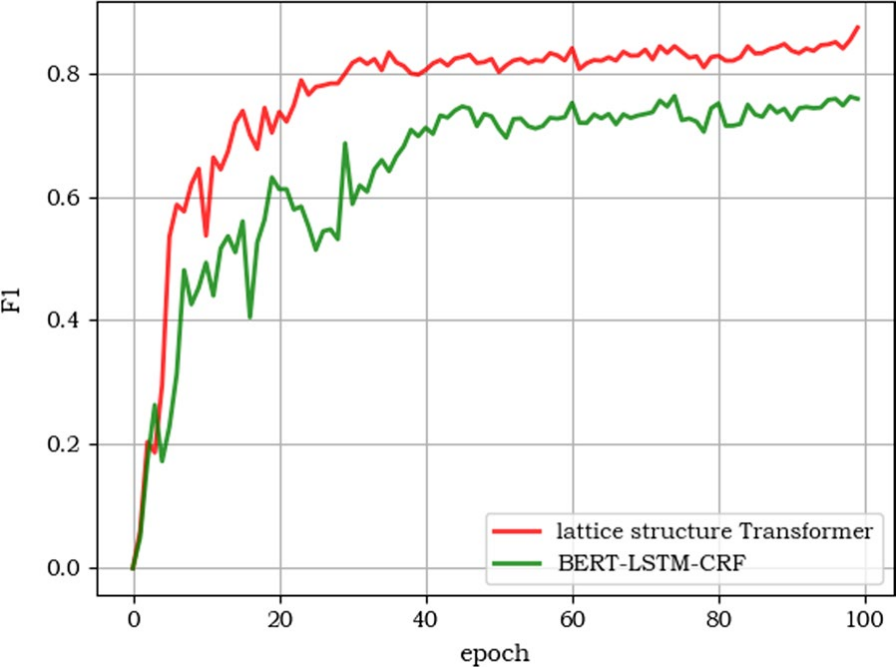
Performance in terms of F1-score with epoch

As shown in Fig. 6, our proposed soft lattice structure Transformer model has a high degree of parallel computing power, allowing our model to converge faster during training (our proposed lattice transformer model begins to converge at approximately 30 epochs, and the BERT-LSTM-CRF model starts to converge at 50 epochs).

To verify the applicability of the model to the extraction of long entities and the recognition of medical-specific abbreviations in Chinese medical records, medical records with more long medical entities (proper nouns longer than 6 characters) and more abbreviations and numbers were purposely selected from the yidu-s4k and Chinese Medical Named Entity Recognition Dataset (MeEE) [34]. In the test set containing more long medical entities, the proportion of long medical entities reached 80%. In the test set containing more abbreviations and numbers, the proportion of abbreviations and numbers reached 70%.

In Table 3, Soft lattice structure Transformer obtains a 91.6% F1-score in long medical entities and performs better at recognizing long entities than at recognizing normal entities.

**Table 3:** Result of models to identify long entities

Models	P	R	F1
LSTM-CRF	82.5	73.4	72.3
GRU-CRF	80.4	72.3	75.7
BERT-CRF	85.4	76.7	76.2
BERT-LSTM-CRF	82.3	83.9	82.6
BERT-GRU-CRF	83.1	84.3	84.2
BERT-FLAT—CRF	85.6	86.2	85.2
BERT-Soft Lattice structure Transformer—CRF	90.5	91.4	91.6

Similarly, the model has a highly competitive performance in recognizing abbreviations and numbers, as shown in Table 4.

**Table 4:** Result of models to identify abbreviations and numbers

Models	P	R	F1
LSTM-CRF	79.54	80.47	80.63
GRU-CRF	80.43	82.35	82.27
BERT-CRF	85.45	86.7	86.25
BERT-LSTM-CRF	86.35	85.9	86.16
BERT-GRU-CRF	86.13	85.3	85.25
Soft Lattice structure Transformer-CRF	90.12	90.72	90.24
BERT-Soft Lattice structure Transformer- CRF	90.65	90.64	90.36

In summary, the proposed lattice structure Transformer-named entity recognition approach has better recognition results and less time consumption.

## Discussion

In recent years, adopting LSTM with CRF has provided excellent results in the NER task. However, to be used in the medical field with clinical relevance, providing explanatory information about text to the model is essential to reduce the computation time and maintain good performance.

In this paper, we devised a model that consists of the BERT module, the lattice structure transformer module, and the CRF module. In combination with the power of BERT and well-designed lattice information, our model has the potential to fully consider lattice information and has robust data parallelism to speed up training.

In previous studies [12], it was indicated that LSTM based on Chinese characters could help to identify clinical entities. Nevertheless, models based on the LSTM network with CRF usually ignore word information and potential words. It can be problematic for Chinese NER if they are not significantly different from these methods of English NER. Some researchers have pointed out that encoding Chinese words in a sentence can reduce the error propagation of segmentation while making use of word information [22].

The soft lattice structure transformer layer not only makes the ability to encode Chinese words to lattice information stronger in our model but can also play the same role as the LSTM in exploring more distinct entity classes in various sentences. Another advantage of the soft lattice structure transformer layer is its high inference efficiency and ease of modeling long-distance dependencies. When recognizing several thousand Chinese characters, there is always a risk that Chinese words are not found in sentences, leading to negative recognition. The Soft Term Position Lattice can avoid this risk.

**Fig. 7:**
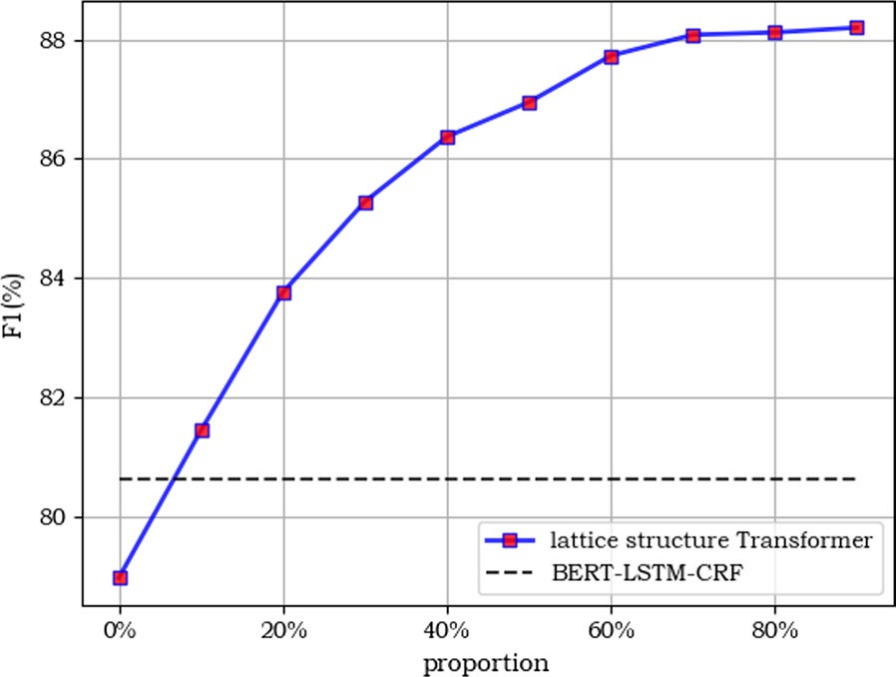
Performance in terms of F1-score with proportions of boundary information

To illustrate the positive effects of introducing entity boundaries using lattice structure and WordPiece, we perform additional experiments to understand our proposed model. Specifically, we design datasets containing different proportions of boundary information and conduct experiments to obtain the results for different proportions of boundary information. As shown in Fig. 7, when the proportion is low, the model performance is lower than that of a traditional LSTM, but when the proportion increases, the model F1-score improves rapidly. Our experiments illustrate that the introduction of information about lexical boundaries is beneficial to improving the performance of the model.

From Fig. 4, we can explain why the BERT in our model was constructed to improve the accuracy of CNER. The reason for improving CNER results is combining the BERT model with LSTM-CRF, which can solve negation and speculation detection issues. In opposition to these approaches, we directly design a special position encoding, Soft Term Position Lattice and transformer to replace LSTM because of the function of lattice structure transformer recognizing uncommon medical entities and parallelization ability. This research pointed out that transformers with soft lattice Chinese word construction can capture potential relevance between Chinese characters and words.

## Conclusions

LSTM has been a long-standing method or one of the components of the NER model, where a core goal is to capture contextual information. Analogously, we presented a different deep learning model that replaces LSTM and has a similar function with less computing. Our experiment used the soft lattice structure Transformer layer to substitute LSTM, illustrating that the lattice structure Transformer layer can reach the performance of LSTM. Notably, the soft lattice structure Transformer model was trained with parallel computing. An especially salient motive for pursuing parallel computing is processing large data while saving time. Moreover, soft lattice structure Transformer with word information can capture more context dependence and relevance.

## Data Availability

The data that support the findings of this study are available from [China Conference on Knowledge Graph and Semantic Computing (CCKS) and Tianchi Data Sets] but restrictions apply to the availability of these data, which were used under license for the current study, and so are not publicly available. Data are however available from the authors upon reasonable request and with permission of [China Conference on Knowledge Graph and Semantic Computing (CCKS) and Tianchi Data Sets].

## Abbreviations

CNER: Clinical named entity recognition
NLP: Natural language processing
NER: Named entity recognition
NES: Named entity segmentation
CRF: Conditional random field
LSTM: Long short-term memory
CNN: Convolution neural network
RNN: Recurrent neural network
BERT: Bidirectional encoder representations from transformers
MeEE: Chinese Medical Named Entity Recognition Dataset
CCKS: China Conference on Knowledge Graph and Semantic Computing
GRU: Gate Recurrent Unit
MLM: Masked language model
NSP: Next sentence prediction
FLAT: Flat-lattice transformer
